# Author Correction: Generalisability of fetal ultrasound deep learning models to low-resource imaging settings in five African countries

**DOI:** 10.1038/s41598-023-30540-z

**Published:** 2023-02-28

**Authors:** Carla Sendra-Balcells, Víctor M. Campello, Jordina Torrents-Barrena, Yahya Ali Ahmed, Mustafa Elattar, Benard Ohene-Botwe, Pempho Nyangulu, William Stones, Mohammed Ammar, Lamya Nawal Benamer, Harriet Nalubega Kisembo, Senai Goitom Sereke, Sikolia Z. Wanyonyi, Marleen Temmerman, Eduard Gratacós, Elisenda Bonet, Elisenda Eixarch, Kamil Mikolaj, Martin Grønnebæk Tolsgaard, Karim Lekadir

**Affiliations:** 1 Departament de Matemàtiques i Informàtica, Universitat de Barcelona, Barcelona, Spain; 2 HP Inc., Barcelona, Spain; 3 Obstetrics and Gynecology Department, School of Medicine, Suez University, Suez, Egypt; 4 Medical Imaging and Image Processing, Center of Informatics Science, Nile University, Sheikh Zayed City , Egypt; 5 Research and Development Division, Intixel, Cairo , Egypt; 6 Department of Radiography, School of Biomedical and Allied Health Sciences, College of Health Sciences, University of Ghana, Accra , Ghana; 7 Division of Midwifery and Radiography, School of Health and Psychological Sciences, University of London, London, UK; 8 Kamuzu University of Health Sciences, Blantyre, Malawi; 9 Department of Electrical Engineering Systems, Laboratory of Engineering System and Telecommunication, University of M’Hamed Bougara Boumerdes, Algiers, Algeria; 10 Obstetrics and Gynecology Department, School of Medicine, Algiers University, Algiers, Algeria; 11 Department of Radiology, Mulago National Referral and Teaching Hospital, Kampala, Uganda; 12 Department of Radiology and Radiotherapy, School of Medicine, Makerere University College of Health Sciences, Kampala, Uganda; 13 Department of Obstetrics and Gynaecology, Aga Khan University Hospital, 3rd Parklands Avenue, Nairobi, Kenya; 14 Centre of Excellence in Women and Child Health, Aga Khan University, Nairobi, Kenya; 15 BCNatal Fetal Medicine Research Center, Hospital Clínic and Hospital Sant Joan de Déu, Universitat de Barcelona, Barcelona, Spain; 16 Institut d’Investigacions Biomèdiques August Pi i Sunyer (IDIBAPS), Barcelona, Spain; 17 Centre for Biomedical Research on Rare Diseases (CIBER-ER), Barcelona, Spain; 18 Barcelona Tech, Universitat Politècnica de Catalunya, Barcelona, Spain; 19 Copenhagen Academy for Medical Education and Simulation and Department of Obstetrics, Rigshospitalet, Copenhagen, Denmark

Correction to: *Scientific Reports* 10.1038/s41598-023-29490-3, published online 15 February 2023

The Funding section in the original version of this Article was incomplete.

“This work received funding from the European Union’s 2020 research and innovation programme under Grant Agreement No. 825903 (euCanSHare project), as well as from the Spanish Ministry of Science, Innovation and Universities under grant agreement RTI2018-099898-B-I00. Additionally, the research leading to these results has received funding from Cerebra Foundation for the Brain Injured Child (Carmarthen, Wales, UK).”

now reads:

“This work received funding from the European Union’s 2020 research and innovation programme under Grant Agreement No. 825903 (euCanSHare project), as well as from the Spanish Ministry of Science, Innovation and Universities under grant agreement RTI2018-099898-B-I00. Additionally, the research leading to these results has received funding from Cerebra Foundation for the Brain Injured Child (Carmarthen, Wales, UK). This research was partly funded by a grant from the European Research Council (ERC) under the European Union’s Horizon Europe research and innovation programme (AIMIX project - grant agreement No. 101044779).”

The original Article has been corrected.

